# The Ferroptosis-Related Noncoding RNA Signature as a Novel Prognostic Biomarker in the Tumor Microenvironment, Immunotherapy, and Drug Screening of Gastric Adenocarcinoma

**DOI:** 10.3389/fonc.2021.778557

**Published:** 2021-11-01

**Authors:** Xinming Chen, Zheng Zhu, Xiaoling Li, Xinyue Yao, Lianxiang Luo

**Affiliations:** ^1^ The First Clinical College, Guangdong Medical University, Zhanjiang, China; ^2^ Department of Medicine, Brigham and Women’s Hospital, Harvard Medical School, Boston, MA, United States; ^3^ Experimental Animal Center, Guangdong Medical University, Zhanjiang, China; ^4^ The Marine Biomedical Research Institute, Guangdong Medical University, Zhanjiang, China; ^5^ The Marine Biomedical Research Institute of Guangdong Zhanjiang, Zhanjiang, China; ^6^ Southern Marine Science and Engineering Guangdong Laboratory, Zhanjiang, China

**Keywords:** ferroptosis, gastric adenocarcinoma, immunotherapy, noncoding RNA, tumor microenvironment

## Abstract

**Background:**

Ferroptosis is a new type of cell death different from apoptosis, necrosis, autophagy, and pyroptosis. This study aimed to explore the relationship between ferroptosis-related noncoding RNA (ncRNA) and gastric adenocarcinoma with regard to immunity and prognosis.

**Methods:**

Ferroptosis-related ncRNA expression profiles and clinical pathology and overall survival information were collected from The Cancer Genome Atlas (TCGA) and the Gene Expression Omnibus database. The ferroptosis-related ncRNA signature was identified by Cox regression analysis and the least absolute shrinkage and selection operator analysis. The survival analysis, receiver operating characteristic (ROC) analysis, and decision curve analysis were adopted to evaluate the prognostic prediction performance of the signature. The correlation between risk and multiple clinical characteristics was analyzed using the chi-square test. The Gene Ontology, Kyoto Encyclopedia of Genes and Genomes, and gene set enrichment analysis were used for mining functions and pathways. The CIBERSORT, ssGSEA, and ESTIMATE algorithms were used to assess immune infiltration and the tumor microenvironment. The response of immunotherapy was predicted using the Submap algorithm, and the Connectivity Map and the ridge regression model were used to screen and evaluate drugs.

**Results:**

A carcinogenic risk signature was constructed using five ferroptosis-related ncRNAs. It showed an extraordinary ability to predict the prognoses of patients with gastric adenocarcinoma [area under the ROC curve (AUC) after 6 years = 0.689; GSE84426, AUC after 6 years = 0.747]. The lower ferroptosis potential level and lower tumor mutation burden were related to the poor prognoses of patients. The high-risk group had more immune cell recruitment, and the overall effect of the anti-immune checkpoint immunotherapy was not as good as that of the low-risk group. The high- and low-risk groups were enriched in tumor- and immune-related pathways, respectively. The screened antitumor drugs, such as genistein, guanabenz, and betulinic acid, improved the survival of the patients.

**Conclusions:**

The ferroptosis-related ncRNA signature is a potential carcinogenic prognostic biomarker of gastric adenocarcinoma.

## Introduction

Gastric cancer is the main gastrointestinal cancer, and the incidence of gastric adenocarcinoma accounts for 95% of gastric cancer (1). The incidence of gastric cancer is often hidden because most of the early symptoms of gastric tumors are not obvious. Most patients are in the middle and late stage when the symptoms become obvious, and the condition is often accompanied by lymph node metastasis. Therefore, developing a model to predict the outcomes of patients is necessary.

Noncoding RNAs (ncRNAs) are RNAs transcribed from genes that do not encode proteins, including but not limited to microRNA, long-noncoding RNA (lncRNA), and circular RNA. ncRNAs have inestimable potential because the biological functions of many ncRNAs have not been clarified. Studies reported that ncRNAs have been critical players in cancer development and progression (2). It is a trend to identify more ncRNAs with therapeutic value in gastric adenocarcinoma.

Ferroptosis is an iron-dependent new programmed cell death form, which is different from apoptosis, cell necrosis, autophagy, and pyroptosis (3). The general mechanism of ferroptosis is the lipid peroxidation of unsaturated fatty acids highly expressed on the phospholipid bilayer, leading to cell death, and ferrous plays a catalytic role (4). Recent studies showed that the expression of some ncRNAs could be regulated to promote ferroptosis in gastric adenocarcinoma cells (5). An extensive analysis is needed to explore the relationship among ferroptosis, ferroptosis-related ncRNA, and gastric adenocarcinoma. Moreover, the possible degree of ferroptosis in the sample should also be measured by the ferroptosis potential index (FPI) (6).

Tumor microenvironment (TME) has always been the focus of gastric adenocarcinoma research, which is closely related to tumor occurrence, growth, and metastasis (7). It reveals the antitumor immune intensity of gastric adenocarcinoma. More in-depth analysis is needed to reveal the relationship between TME and gastric adenocarcinoma.

Immune checkpoints, such as programmed cell death protein 1 (PD-1) and CTLA4, can downregulate the immune state of the body. The immune checkpoint inhibitor immunotherapy of tumor mainly refers to a new treatment method of using PD-1 and CTLA4 inhibitors to enhance the immune function of the body by increasing the antitumor activity of T cells and achieve the purpose of eliminating the tumor (8). The responsiveness of immune checkpoint inhibitors is measured by the tumor immune dysfunction and exclusion (TIDE) score (9, 10).

However, a comprehensive analysis of tumor immune microenvironment and immunotherapy based on ferroptosis-related ncRNA prognostic biomarkers has not been conducted. In this study, a ferroptosis-related ncRNA signature and a matching nomogram were developed. This ferroptosis-related ncRNA prognostic signature could effectively predict the prognoses of patients with gastric adenocarcinoma. It was conducive to tumor immune checkpoint inhibitor immunotherapy and the identification of immune components in the tumor immune microenvironment. In addition, this ferroptosis-related ncRNA prognostic signature was adopted to screen anticancer drugs to reduce the risk and improve the prognoses of patients with gastric adenocarcinoma and predict the half-maximal inhibitory concentration (IC_50_) of the drugs.

## Materials and Methods

### Data Acquisition and Processing

RNA sequencing data (fragments per kilobase of exon model per million mapped fragments) of stomach adenocarcinoma (STAD) and matching clinical data were downloaded from The Cancer Genome Atlas (TCGA) database (https://portal.gdc.cancer.gov/) and Gene Expression Omnibus database (GEO; https://www.ncbi.nlm.nih.gov/geo/). Among them, the data from the TCGA database included the normal samples also. The training cohort was from the TCGA-STAD dataset, and the validation cohort was from the GSE84426 dataset. The RNAs of the two cohorts were intersected by Ensembl ID (https://asia.ensembl.org/index.html) to obtain the common RNAs. In addition, the human gene annotation file downloaded from the National Center for Biotechnology Information (ftp://ftp.ncbi.nlm.nih.gov/genomes/) was used to classify protein-coding genes and ncRNAs using the R packages “rtracklayer” and “SummarizedExperiment.” Then, ferroptosis-related genes gathered from FerrDb (http://www.zhounan.org/ferrdb/) were identified. Subsequently, ferroptosis-related ncRNAs were obtained through the correlation analysis of ferroptosis-related genes and ncRNAs using the R language (Wilcoxon test, *P* < 0.05, *|cor|* > 0.2). Meanwhile, ferroptosis-related genes with a strong correlation with ferroptosis-related ncRNAs were also collected. The tumor mutation burden (TMB) file of STAD was derived as described by Thorsson (11). The TMB was defined as the number of mutated bases per one million bases. The single-sample gene set enrichment analysis (ssGSEA) in the R software package “gsva” was used to calculate the enrichment score of positive or negative regulation of ferroptosis by positively regulated ferroptosis-related genes (NOS2, NOX1, CP, AURKA, MIOX, TRIB3, MYB, HELLS, GDF15, NOX3, ALOX15, and ALB) and negatively regulated ferroptosis-related genes (TP63, ALOX12, PLIN4, ANGPTL7, AKR1C1, and HBA1), respectively, to dissect the ferroptosis levels of the TCGA-STAD samples. The normalized difference between positive and negative components was defined as the FPI (6). The TIDE algorithm (http://tide.dfci.harvard.edu/) was used to calculate the TIDE score of each sample to predict the response of anti-PD-1 and anti-CTLA4 immunotherapy in each sample of the TCGA-STAD cohort (9, 10). If the TIDE score of the sample is greater than 0, the sample does not respond to the immune checkpoint inhibitors; if it is less than 0, the sample responds to the immune checkpoint inhibitors. The clinical characteristics of the GSE84426 cohort in the present study include overall survival time, survival status, age, gender, T stage, and N stage. In addition to the aforementioned clinical characteristics, the TCGA-STAD cohort also includes grade, M stage, FPI, TMB, and TIDE. The tumor samples from both cohorts included patients of all stages. All tumor samples were retained for univariate Cox analysis and the least absolute shrinkage and selection operator (LASSO) regression model construction. After construction of the LASSO regression model, samples with incomplete clinical information and samples with some clinical information that cannot be calculated were excluded. The rest of the samples were retained for subsequent analyses. The data of TCGA-STAD and GSE84426 downloaded for genomic analysis were from public databases. TCGA-STAD used Ensembl ID (https://asia.ensembl.org/index.html) and GSE84426 used “ILMN Gene” (https://www.ncbi.nlm.nih.gov/geo/query/acc.cgi?acc=GPL6947). The IDs of both databases will be converted to the same gene symbol names.

### Preparation of Immune Analyses

ssGSEA was performed to calculate the scores of 16 immune-infiltrating cells and 13 immune-related pathways in each TCGA-STAD sample using the R software package “gsva”. The CIBERSORT algorithm was also adopted through the R language to evaluate the immune cells. Each TCGA-STAD sample corresponded to a *P*-value. If the *P*-value was less than 0.05, the result of immune infiltration of the sample was reliable. In the immune analyses, samples with a *P*-value less than 0.05 were only retained in the present study. The R software package “estimate” was used to calculate the stromal score, immune score, and ESTIMATE score of each TCGA-STAD sample.


ESTIMATE Score=stromal score+immune score

The higher the stromal score (immune score) of the sample, the higher the content of the stromal cells (immune cells).

### Identification of Ferroptosis-Related ncRNAs With Prognostic Value and Unsupervised Class Discovery

A univariate Cox regression analysis of ferroptosis-related ncRNAs was performed to obtain ferroptosis-related ncRNAs with the prognostic value using the R language. The input file was the tumor tissue sample of the training cohort. The *P*-value filter was set to 0.1 while maintaining the *P*-value of two of the five ncRNAs with a prognostic value less than 0.05 so as to ensure that enough ncRNAs with the prognostic value were obtained by univariate Cox regression analysis. The other three ncRNAs with a *P*-value between 0.05 and 0.1 were capable of being regarded as having a prognostic value and could be used for the construction of the signature. Similarly, the survival analyses (overall survival, OS) of ncRNAs were carried out according to the optimal *P*-value cutoff. The R software package “corplot” was used to explore the correlation of immune checkpoint genes (PD-1, PD-L1, and CTLA4) and ferroptosis-related ncRNAs in expression. Then, unsupervised class discovery analysis was conducted using the R software package “ConsensusClusterPlus.” The input file was the expression matrix of prognosis-related ncRNAs, normalized and standardized by the “scale” function of the R language. The algorithm was “km”, and the distance calculation method was “Euclidean”. The optimal *K* value was determined according to a series of output diagrams. How many classes the samples of TCGA-STAD were divided into depended on the optimal *K* value. The Kaplan–Meier curves of OS were generated using the R software packages “survival” and “survminer” to reflect the survival difference of clusters (*P* < 0.05).

### Development and Preliminary Validation of a Ferroptosis-Related ncRNA Signature

The LASSO regression analysis of ferroptosis-related ncRNAs with the prognostic value was performed to obtain the coefficient of each ncRNA using the R language. The risk score of each sample was equal to the sum of ncRNA expression multiplied by the corresponding coefficient. According to the median of the risk score, the TCGA-STAD samples were divided into high- and low-risk groups. Similarly, the risk score of each sample in the GSE84426 validation cohort was calculated through previously obtained coefficients for each gene and divided into high- and low-risk groups according to the median of its own risk score. The survival analysis of OS was carried out to explore the prognostic value of the signature, and the receiver operating characteristic (ROC) curve analysis was used to estimate the predictive practicability of the signature. The aforementioned analyses included both TCGA-STAD and GSE84426 cohorts, which were completed using the R software.

### Analysis of Correlation in Ferroptosis-Related ncRNA Signature and Various Clinical Characteristics

Clinical correlation analyses of the TCGA-STAD and GSE84426 cohorts were conducted to determine whether differences existed in clinical characteristics between the high- and low-risk groups (chi-square test, *P* < 0.05). The clinical characteristics of the TCGA-STAD cohort comprised age, gender, grade, stage, T stage, M stage, N stage, FPI, TIDE, TMB, immune score, and ESTIMATE score. Similarly, the clinical characteristics of the GSE84426 cohort comprised age, gender, stage, T stage, and N stage. Each clinical characteristic was allocated into two groups. Heat maps were drawn using the R software package “pheatmap” to show the differential expression analysis and clinical correlation analysis of the high- and low-risk groups at the same time. Although cluster was also in the heat map of TCGA-STAD cohort, it was not included in the clinical correlation analysis (or in the chi-square test).

Univariate and multivariate Cox regression analyses of clinical characteristics were performed on the two cohorts in which the GSE84426 cohort was adopted as the validation set. The analyses included the risk score, gender, FPI, age, grade, T stage, N stage, and M stage. However, the clinical characteristics of the GSE84426 cohort did not include FPI, grade, and M stage. The ROC curve analyses of the clinical characteristics were conducted to compare the performance of each clinical characteristic. The decision curve analysis (DCA) was also performed using R software packages “survival” and “ggDCA” to better demonstrate the prognostic prediction performance of the different clinical characteristics of the TCGA-STAD and GSE84426 cohorts. In the decision curve graph, the straight line “*y* = 0” assumed that all samples were negative, the other curve representing “all” assumed that all samples were positive, and several other curves represented clinical characteristics and the risk score. The farther the other curves were from the curve representing “all,” the higher the accuracy of this factor in predicting patient survival. This study included a “combined” curve that combined all clinical characteristics.

The FPI and risk score were combined for the survival analysis of OS, and the TMB was the same. Furthermore, a nomogram containing clinical characteristics with the *P*-value less than 0.05 in the multivariate regression analysis of TCGA-STAD cohort was constructed by R software packages “survival” and “regplot.” The method of the nomogram used was to add the scores corresponding to the clinical characteristics included in the nomogram to obtain the total points so as to predict the probability that the patient survival time was less than 1, 3, and 5 years. Then, the R software package “rms” was used to draw the calibration curves of 1, 3, and 5 years to verify the nomogram. The methods used were “km” and “boot.” The calculation was repeated with 100 samples in each group. The better the calibration curves and diagonal fit, the more reliable the multivariate Cox regression analysis.

### Screening of Hub Genes, Functional Enrichment Analyses, and Pathway Enrichment Analyses

In the TCGA-STAD cohort, the ferroptosis-related genes obtained from the previous co-expression analysis were co-expressed with the ferroptosis-related ncRNAs involved in the signature, and the key genes were obtained. The differential expression of these key genes was analyzed by the R software package “limma” based on normal tissue *versus* tumor tissue and high-risk group *versus* low-risk group (false discovery rate, FDR, < 0.05]. Log_2_(fold change) was not filtered, and the hub genes closely related to the prognosis signature were obtained. Protein–protein interaction network analysis (https://string-db.org/cgi/input.pl), correlation network analysis using the R software package “igraph” (cutoff = 0.42), and co-expression network rendering with the ferroptosis-related ncRNAs involved in the signature using the Cytoscape software were carried out for these hub genes, which closely related to the prognosis signature.

Gene Ontology (GO) and Kyoto Encyclopedia of Genes and Genomes (KEGG) enrichment analyses based on the hub genes were performed using the R software package “clusterProfiler” (FDR < 0.05) (12). The GO enrichment analysis divided the gene functions into three parts: cellular component, molecular function, and biological process. The gene set enrichment analysis (GSEA) was achieved using the GSEA software (http://www.gsea-msigdb.org/gsea/index.jsp) to explore the enriched KEGG pathways between the high- and low-risk groups of the TCGA-STAD cohort (FDR < 0.05 or *P* < 0.05). Multiple GSEA diagrams were generated using the R software packages “grid”, “gridExtra”, “ggplot2”, and “plyr”.

### Analyses of Immune Cells, Immune-Related Pathways, TME, and Immune Checkpoints

After the preparation of immune analyses, cluster differential analyses of immune cells, stromal score, immune score, and ESTIMATE score were performed based on the results of unsupervised class discovery, CIBERSORT, and ESTIMATE (using the R software package “limma”, *P* < 0.05). The R software package “vioplot” was used to visualize the differential analysis results of immune cells in the high- and low-risk groups. The R software packages “limma”, “ggpubr”, and “reshape2” were adopted to visualize the results of ssGSEA and ferroptosis-related ncRNA signature (score differential analysis of immune cells and immune-related pathways in the high- and low-risk groups). Based on the results obtained from CIBERSORT and ferroptosis-related ncRNA signature, the Spearman correlation diagrams of various immune cells and risk score were generated using the R software packages “limma”, “ggplot2”, “ggpurb”, and “ggExtra” (*P* < 0.05). An immune cell infiltration estimation file for TCGA was downloaded from the TIMER2.0 database (http://timer.cistrome.org/), and R software packages “limma” and “pheatmap” were used to analyze the immune cell infiltration difference between the high- and low-risk groups (*P* < 0.05) and visualize the results for validation. The differential expression of immune checkpoint genes (CTLA4, PD-1, and PD-L1) in the high- and low-risk groups was analyzed using the R software package “limma”. Moreover, the list of immune genes was downloaded from the Tracking Tumor Immunophenotype (TIP) (http://biocc.hrbmu.edu.cn/TIP/index.jsp) metaserver. The negative regulatory immune genes were separated out. The differential expression of the high- and low-risk groups was analyzed using the heat map (by R software packages “limma” and “pheatmap”, *P* < 0.05). Negative regulatory immune genes referred to genes that downregulate the immune response.

### Immunotherapy, Drug Screening, and Drug Prediction

After obtaining the TIDE score of each sample of the TCGA-STAD cohort in data acquisition and processing, the Submap algorithm of GenePattern (https://cloud.genepattern.org/gp/pages/index.jsf) was used to predict the response of immunotherapy in the high- and low-risk groups (nominal *P <*0.05 or Bonferroni correction <0.05). In this step, the information file of 47 immunotherapy samples and the gene expression file of 47 immunotherapy samples in the study of Lu were used (13). Then, a comparative heat map was drawn using the R software package “pheatmap”.

The ferroptosis-related genes obtained from the differential expression analysis of the high- and low-risk groups (as explained above) were used for the Connectivity Map (CMAP) drug screening (enrichment < 0, *P* < 0.05; https://portals.broadinstitute.org/cmap/). “Enrichment” less than 0 meant that this drug was conducive to inhibit the expression of high-risk genes, reduce the risk of patients, and improve the survival rate of patients. A *P*-value less than 0.05 was considered notable enrichment. Then, the structure of the screened drugs was drawn using the ChemDraw software.

The ridge regression models were constructed using the PRRophetic algorithm according to the list of drugs contained in the Genomics of Drug Sensitivity in Cancer (GDSC) database (https://www.cancerrxgene.org/) (13). The TCGA-STAD gene expression profile and the high- and low-risk grouping information were used in the R software package “PRRophetic” to predict the IC_50_ of the drugs. The smaller the IC_50_ value of the drug, the stronger the ability of the drug to inhibit cell growth, and the more effective it is in treating cancer.

### Statistical Analysis

Statistical analyses were completed using the R software, version 4.0.3. The Wilcoxon test was used in the co-expression analysis of ferroptosis-related RNAs (mRNAs and ncRNAs) and differential analyses. The chi-square method was used in clinical correlation analyses. The Fisher method was used in the differential analysis of TIDE scores in the high- and low-risk groups. The Spearman method was used in the correlation analyses of the risk score and scores of immune cells. A *P*-value <0.05 was considered statistically significant.

## Results

### Screening of Ferroptosis-Related ncRNAs With a Prognostic Value in Gastric Adenocarcinoma

A total of 373 samples were present in the TCGA-STAD cohort, including 30 normal samples and 343 tumor samples. If normal samples, samples with incomplete clinical information, and samples with some clinical information that cannot be calculated were excluded, 269 samples with the most complete clinical information remained (**Table 1**). Moreover, 76 tumor samples were present in the GSE84426 cohort.

**Table 1:** Clinical characteristics of the stomach adenocarcinoma patients included in this study.

Characteristics	TCGA-STAD (*n* = 269)	GSE84426 (*n* = 76)
Age		
≤65	118 (43.87%)	22 (28.95%)
>65	151 (56.13%)	54 (71.05%)
Gender		
Male	166 (61.71%)	54 (71.05%)
Female	103 (38.29%)	22 (28.95%)
Grade		
G1	5 (1.86%)	–
G2	93 (34.57%)	–
G3	171 (63.57%)	–
Stage		
I	37 (13.75%)	–
II	87 (32.34%)	–
III	118 (43.87%)	–
IV	27 (10.04%)	–
T stage		
T1	13 (4.83%)	0
T2	55 (20.45%)	3 (3.95%)
T3	133 (49.44%)	25 (32.90%)
T4	68 (25.28%)	48 (63.15%)
M stage		
M0	253 (94.05%)	–
M1	16 (5.95%)	–
N stage		
N0	85 (31.6%)	9 (11.84%)
N1	68 (25.28%)	33 (43.42%)
N2	63 (23.42%)	33 (43.42%)
N3	53 (19.7%)	1 (1.32%)
FPI		
≤0	108 (40.15%)	–
>0	161 (59.85%)	–
TIDE score		
<0	84 (31.23%)	–
>0	185 (68.77%)	–

STAD, stomach adenocarcinoma; FPI, ferroptosis potential index; TIDE, tumor immune dysfunction and exclusion.

**Figure 1:**
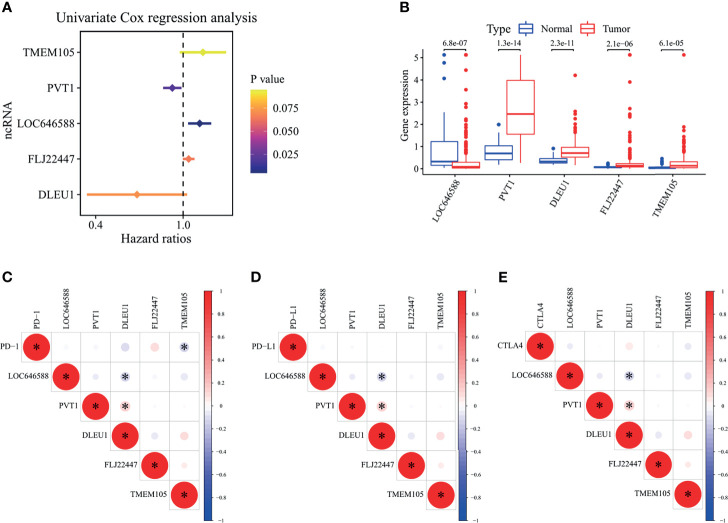
Analysis of univariate Cox regression, differential ncRNA expression profiles, and RNA correlation analysis in The Cancer Genome Atlas—stomach adenocarcinoma cohort. **(A)** Forest plot showing the *P*-values and hazard ratios of five ferroptosis-related ncRNAs from univariate Cox regression analysis. A hazard ratio greater than 1 indicates that the ncRNA is a risk factor for the survival of gastric adenocarcinoma patients, and a hazard ratio less than 1 indicates that the ncRNA is a protective factor for the survival of gastric adenocarcinoma patients. **(B)** Box plot for ferroptosis-related ncRNAs generated by a comparison of the normal group *versus* the tumor group. The value shown in the figure is the *P*-value. **(C–E)** Correlation among three immune checkpoint genes (PD-1, PD-L1, and CTLA4) and ferroptosis-related ncRNAs. Red indicates a positive correlation, blue indicates a negative correlation, and an asterisk indicates a significant correlation between the two RNAs. STAD, stomach adenocarcinoma. *P < 0.05.

The RNAs of the TCGA-STAD and GSE84426 samples were intersected to obtain 15,302 common RNAs by Ensembl ID. This study identified 160 ncRNAs from these RNAs, and the rest were mRNAs and unrecognized ncRNAs. By the correlation analysis of 269 ferroptosis-related genes and 160 ncRNAs, 52 ferroptosis-related ncRNAs and 220 ferroptosis-related genes with a strong correlation with ferroptosis-related ncRNAs were screened. Five prognostic ncRNAs (TMEM105, PVT1, LOC646588, FLJ22447, and DLEU1) were screened by univariate Cox regression analysis (**Figure 1A**). TMEM105, LOC646588, and FLJ22447 were high-risk ncRNAs in the TCGA-STAD samples (hazard ratio, HR > 1). Their high expression predicted poor prognoses. On the contrary, PVT1 and DLEU1 were low-risk ncRNAs (HR < 1). The specific outputs are shown in **Table 2**. The results of the survival curve analysis verified the results of the univariate Cox analysis: the high expression of TMEM105, LOC646588, and FLJ22447 predicted the poor prognosis of patients. The high expression of DLEU1 and PVT1 indicates a good prognosis (**Supplementary Figures S1A–E**). Except LOC646588, the expression of four other ncRNAs was upregulated in tumor tissues (**Figure 1B**). Among the three immune checkpoint genes (PD-1, PD-L1, and CTLA4), PD-1 and LOC646588 correlated negatively with TMEM105 and DLEU1, respectively, and PVT1 correlated positively with DLEU1 (**Figures 1C–E**). The results suggested that TMEM105, PVT1, FLJ22447, DLEU1, and LOC646588 closely related to the prognoses of patients with gastric adenocarcinoma. In addition to the mutual regulation relationship between the five ncRNAs, TMEM105 also negatively regulated the expression of PD-1.

**Table 2:** Results of univariate Cox analysis and LASSO analysis.

ncRNA	Univariate Cox		Coefficient
	HR (95%CI)	*p*	
LOC646588	1.189 (1.054–1.342)	0.005	0.129555727389162
PVT1	0.893 (0.811–0.983)	0.020	-0.00441041093790246
DLEU1	0.617 (0.365–1.043)	0.071	-0.193535787611945
FLJ22447	1.060 (0.997–1.127)	0.064	0.148181925600644
TMEM105	1.231 (0.965–1.569)	0.095	0.0363808906413759

LASSO, least absolute shrinkage and selection operator; ncRNA, noncoding RNA; HR, hazard ratio; CI, confidence interval.

### Unsupervised Class Discovery to Construct a Novel Grouping System

**Figure 2:**
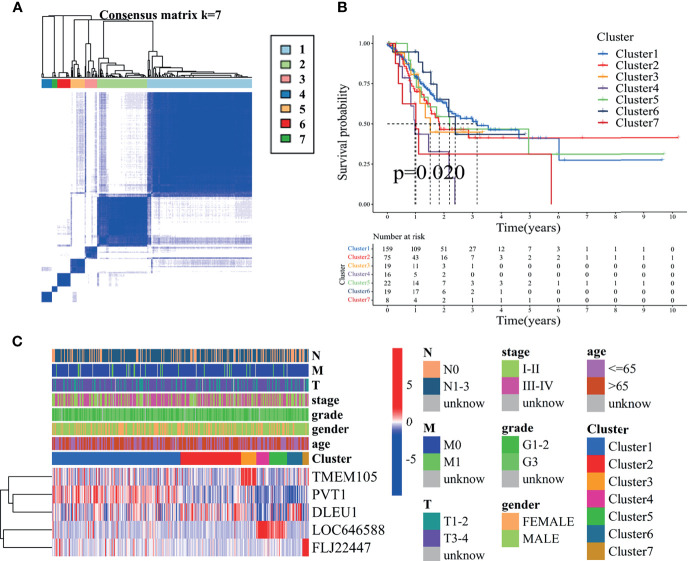
Consensus clustering for five ferroptosis-related ncRNAs in The Cancer Genome Atlas—stomach adenocarcinoma (TCGA-STAD) cohort. **(A)** Consensus clustering plot showing that 7 was the optical *K* value. The purer the color, the more appropriate the grouping and the more obvious the separation. **(B)** Kaplan–Meier curves for the OS of patients from TCGA-STAD cohort with the classes (*P* < 0.05). They mean that, with the extension of time, the survival possibility of patients gradually decreases. **(C)** Heat map for ferroptosis-related ncRNAs generated by comparison of clusters and clinical characteristics. This heat map contains the expression levels of five ncRNAs in seven clusters. The greater the proportion of red in a region, the higher the expression. OS, overall survival.

Through unsupervised class discovery analysis of prognosis-related ncRNAs, the optimal *K* value obtained was 7 (**Figure 2A** and **Supplementary Figure S2A**). The TCGA-STAD samples were assigned into seven clusters. The prognoses of clusters 4 and 7 were the worst, and those of clusters 1 and 6 were the best (*P* = 0.02) (**Figure 2B**). The expression of five ncRNAs in clusters is shown in **Figure 2C**. TMEM105 was highly expressed in cluster 3, PVT1 in cluster 1, DLEU1 in clusters 1, 2, and 6, LOC646588 in clusters 4 and 5, and FLJ22447 in cluster 7. The expression of PD-1, PD-L1, and CTLA4 in clusters 1 and 2 was notably higher than that in almost all other clusters (**Supplementary Figures S2B–D**). The aforementioned results indicated great differences in the survival of the seven clusters, and the three immune checkpoint genes were significantly differentially expressed in the seven clusters. The unsupervised class discovery refined the classification of gastric adenocarcinoma.

### Identification and Validation of the Ferroptosis-Related ncRNA Signature, Clinical Correlation Analysis, and Independent Prognostic Factor Analysis

A signature of five ferroptosis-related ncRNAs was identified. The coefficients are shown in **Table 2**. The risk score of each sample was calculated as follows:


$$\text{Risk score}=\sum_{i=1}^{5}\left(\text{Cofficient}(i\right)*\text{Expr}(i))$$

**Figure 3:**
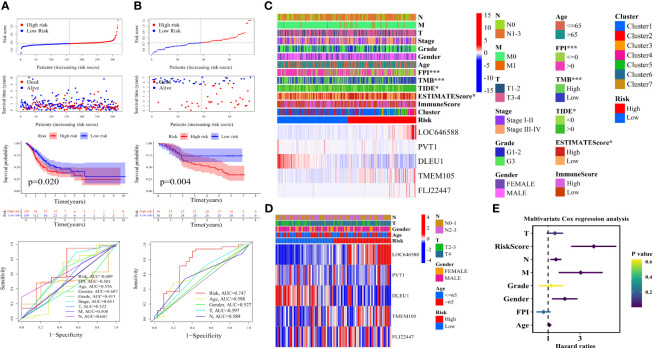
Construction of ferroptosis-related ncRNA signature, clinical correlation analysis, and independent prognosis analysis. Grouped according to the median value of risk score, distribution of survival status and risk score, Kaplan–Meier survival curves, and clinically relevant ROC curves of **(A)** The Cancer Genome Atlas—stomach adenocarcinoma (TCGA-STAD) cohort and **(B)** the GSE84426 cohort. Correlation analysis of clinical characteristics by chi-square test and heat maps of five ferroptosis-related ncRNAs of **(C)** TCGA-STAD cohort and **(D)** the GSE84426 cohort. **(E)** Forest plot showing the *P*-values, hazard ratios of the clinical characteristics of TCGA-STAD from multivariate Cox regression analysis. ROC, receiver operating characteristic. *P < 0.05; ***P < 0.001.

As shown in **Figure 3A**, the tumor samples were divided into two groups according to the median value of the risk score. The greater the risk score, the more the patients died. The survival of the high-risk group was significantly worse than that of the low-risk group in the TCGA-STAD training cohort (*P* = 0.020). The risk AUC value in the ROC analysis of 6 years reached 0.689, which was higher than the AUC values of other clinical characteristics. The GSE84426 cohort was validated for survival analysis and ROC analysis (*P* = 0.004 and risk AUC = 0.747) (**Figure 3B**).

As shown in **Figure 3C**, LOC646588, TMEM105, and FLJ22447 were upregulated in the high-risk group, and PVT1 and DLEU1 were upregulated in the low-risk group. The samples with FPI greater than 0, high TMB, TIDE less than 0, and low ESTIMATE score were significantly concentrated in the low-risk group, and the samples with FPI less than or equal to 0, low TMB, TIDE greater than 0, and high ESTIMATE score were significantly concentrated in the high-risk group (^***^ *P* < 0.001 and ^*^ *P* < 0.05). Except for clusters 1, 2, and 6, the other clusters were mainly distributed in the high-risk group. Clusters 4 and 5 had the highest risk score (**Figure 3C**). The GSE84426 cohort confirmed the expression of five ncRNAs (**Figure 3D**). The detailed clinical correlation analysis results are shown in **Table 3**.

**Table 3:** Risk based chi-square test of clinical characteristics.

Covariates	Risk	Total	High	Low	Chi	*P*-value
Age	≤65	118 (43.87%)	62 (46.97%)	56 (40.88%)	0.7814	0.376698
Age	>65	151 (56.13%)	70 (53.03%)	81 (59.12%)		
Gender	Female	103 (38.29%)	49 (37.12%)	54 (39.42%)	0.0685	0.793606
Gender	Male	166 (61.71%)	83 (62.88%)	83 (60.58%)		
Grade	G1–2	98 (36.43%)	49 (37.12%)	49 (35.77%)	0.0108	0.917084
Grade	G3	171 (63.57%)	83 (62.88%)	88 (64.23%)		
Stage	Stages I–II	124 (46.1%)	58 (43.94%)	66 (48.18%)	0.3299	0.565703
Stage	Stages III–IV	145 (53.9%)	74 (56.06%)	71 (51.82%)		
T	T1–2	68 (25.28%)	31 (23.48%)	37 (27.01%)	0.2748	0.600126
T	T3–4	201 (74.72%)	101 (76.52%)	100 (72.99%)		
M	M0	253 (94.05)	122 (92.42)	131 (95.62)	0.7228	0.395234
M	M1	16 (5.95%)	10 (7.58%)	6 (4.38%)		
N	N0–1	153 (56.88)	75 (56.82%)	78 (56.93%)	0	1
N	N2–3	116 (43.12)	57 (43.18%)	59 (43.07%)		
FPI	≤0	108 (40.15)	73 (55.3%)	35 (25.55%)	23.5476	1.00E-06
FPI	>0	161 (59.85)	59 (44.7%)	102 (74.45%)		
TIDE	<0	84 (31.23%)	32 (24.24%)	52 (37.96%)	5.266	0.021746
TIDE	>0	185 (68.77%)	100 (75.76	85 (62.04%)		
TMB	High TMB	135 (50.19%)	51 (38.64%)	84 (61.31%)	12.937	0.000322
TMB	Low TMB	134 (49.81%)	81 (61.36%)	53 (38.69%)		
ESTIMATE score	High	134 (49.81%)	75 (56.82%)	59 (43.07%)	4.5507	0.032905
ESTIMATE score	Low	135 (50.19%)	57 (43.18%)	78 (56.93%)		
Immune score	High	134 (49.81%)	66 (50%)	68 (49.64%)	0	1
Immune score	Low	135 (50.19%)	66 (50%)	69 (50.36%)		

TMB, tumor mutation burden; FPI, ferroptosis potential index; TIDE, tumor immune dysfunction and exclusion.

The analysis of the independent prognostic factors of the TCGA-STAD samples showed that the risk score, N stage, M stage, gender, and age could effectively predict the prognoses of patients with gastric adenocarcinoma (**Supplementary Figure S2E** and **Figure 3E**). The FPI was a protective factor for the risk of gastric adenocarcinoma (HR < 1), and the other clinical characteristics of the analysis were hazard factors (HR > 1). The analysis of independent prognostic factors of the GSE84426 cohort showed that age and risk score were independent prognostic factors (**Supplementary Figures S2F, G**). The risk score was greater than the other clinical characteristics irrespective of the training or validation cohort in predicting prognose. Detailed independent prognostic analysis results are shown in **Tables 4** and**5**.

**Table 4:** Cox regression analyses of clinical characteristics and survival in The Cancer Genome Atlas— stomach adenocarcinoma.

Characteristics	Univariable Cox		Multivariable Cox	
	HR (95%CI)	*p*	HR (95%CI)	*p*
FPI	0.929 (0.750–1.152)	0.502	0.856 (0.668–1.096)	0.217
Age	1.024 (1.005–1.043)	0.014	1.043 (1.022–1.066)	8E-05
Gender	1.613 (1.062–2.451)	0.025	1.775 (1.144–2.755)	0.010
Grade	1.258 (0.861–1.839)	0.236	1.108 (0.731–1.681)	0.629
T	1.282 (1.003–1.639)	0.047	1.263 (0.951–1.676)	0.106
M	1.861 (0.937–3.695)	0.076	3.073 (1.440–6.554)	0.004
N	1.359 (1.143–1.617)	0.001	1.310 (1.084–1.583)	0.005
Risk score	4.675 (2.324–9.405)	2E-05	4.810 (2.212–10.461)	7E-05

HR, hazard ratio; CI, confidence interval; FPI, ferroptosis potential index.

**Table 5:** Cox regression analyses of clinical characteristics and survival in GSE84426.

Characteristics	Univariable Cox		Multivariable Cox	
	HR (95%CI)	*p*	HR (95%CI)	*p*
Age	1.023 (0.993–1.053)	0.132	1.031 (1.000–1.063)	0.048
Gender	1.167 (0.547–2.493)	0.689	1.075 (0.484–2.386)	0.859
T	1.698 (0.873–3.304)	0.119	1.408 (0.687–2.885)	0.351
N	1.465 (0.876–2.450)	0.146	1.654 (0.944–2.898)	0.078
Risk score	29.78 (3.023–293.24)	0.004	25.82 (2.051–325.35)	0.012

HR, hazard ratio; CI, confidence interval.

The aforementioned results suggested that FPI, TMB, TIDE, and ESTIMATE score significantly correlated with high and low risks, and the risk score was the optimal independent prognostic factor. The five-ncRNA risk signature effectively predicted the survival of patients with gastric adenocarcinoma and correlated with multiple clinical characteristics.

### Construction of Nomogram, DCA, Correlation With FPI, and Correlation With TMB to Analyze the Ferroptosis-Related ncRNA Signature and Clinical Characteristics

**Figure 4:**
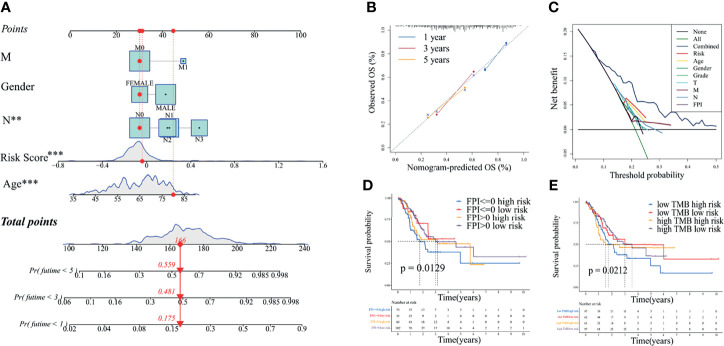
Comprehensive analysis of excellent independent prognostic factors in The Cancer Genome Atlas—stomach adenocarcinoma cohort. **(A)** Nomogram constructed by independent prognostic factors with *P <*0.05 in the multivariate Cox regression analysis. **(B)** Nomogram calibration curves for 1, 3, and 5 years. **(C)** Decision curves used to compare the accuracy of multiple models in predicting the survival of patients with gastric adenocarcinoma. “Combined” represents the model jointly constructed by all clinical characteristics in the figure. **(D)** Joint survival curves of the FPI and risk. **(E)** Joint survival curves of the TMB and risk. FPI, ferroptosis potential index; TMB, tumor mutation burden. ^**^ *P* < 0.01; ^***^ *P* < 0.001.

A nomogram containing significant clinical characteristics in multivariate Cox regression analysis was constructed to explore further the potential of ferroptosis-related ncRNA prognostic signature and clinical characteristics in predicting the prognoses of patients with gastric adenocarcinoma. As shown in **Figure 4A**, the N stage (^**^ *P* < 0.01), age (^***^ *P* < 0.001), and risk score (^***^ *P* < 0.001) could independently predict the prognoses of patients. The calibration curves verified the reliability of the nomogram (**Figure 4B**). The DCA showed that, in most cases, the risk signature had the highest accuracy in predicting the 1-year survival of the TCGA-STAD cohort (**Figure 4C** and **Supplementary Figure S3A**). The accuracy of the risk signature in predicting the 5-year survival of the GSE84426 cohort was higher than that of other clinical characteristics (**Supplementary Figures S3B, C**). The 1-year survival DCA results of the GSE84426 cohort could not be obtained due to insufficient effective sample size.

As shown in **Supplementary Figure S3D**, the FPI in tumor tissue was much higher than that in normal tissue for the TCGA-STAD cohort (*P* = 8.3e-17). Consistent with the results of the clinical correlation analysis, the FPI in tumor tissues in the high-risk group was significantly lower than that in the low-risk group (*P* = 5.5e-08) (**Supplementary Figure S3E**), indicating that a high ferroptosis level was conducive to reducing the risk of patients with gastric adenocarcinoma. As shown in **Figure 4D**, survival analysis (OS) suggested that, for the high-risk group, the FPI greater than 0 predicted better survival than the FPI less than or equal to 0. It was not notable for the low-risk group.

Similar to the results of the clinical correlation analysis, the TMB was much higher in the low-risk group than in the high-risk group (*P* = 8.3e-08) (**Supplementary Figure S3F**). The high TMB was beneficial to reduce the risk of gastric adenocarcinoma. As shown in **Figure 4E**, the survival analysis (OS) of the TCGA-STAD cohort suggested that, for the high-risk group, the high TMB predicted better survival than the low TMB over 2 years. It was not obvious for the low-risk group.

These results indicated that the ferroptosis-related ncRNA risk signature had the highest accuracy in predicting patient survival. The high FPI and the high TMB were conducive to reducing the risk of patients with gastric adenocarcinoma.

### Screening of Ferroptosis-Related Hub Genes, Functional Enrichment Analysis, and Pathway Enrichment Analysis

After the co-expression analysis and differential expression analyses based on normal tissue *versus* tumor tissue and high-risk group *versus* low-risk group, 50 ferroptosis-related hub genes closely related to prognostic signature were obtained in the TCGA-STAD cohort. Protein interaction was observed between ferroptosis-related hub genes transforming growth factor beta receptor 1 (TGFBR1) and caveolin 1 (CAV1) (**Supplementary Figure S4A**). The expression of TGFBR1 (CAV1) and alpha-B crystallin (CRYAB) correlated positively (**Supplementary Figure S4B**). TGFBR1 was co-expressed with ferroptosis-related ncRNA LOC646588 (**Supplementary Figure S4C**). CAV1 was co-expressed with ncRNA LOC646588 and ncRNA FLJ22447 (**Supplementary Figure S4C**). GABA type A receptor-associated protein like 1 (GABARAPL1) correlated positively with the expression of CRYAB (**Supplementary Figure S4B**), and co-expression relationships were found between GABARAPL1 (CRYAB) and ncRNA LOC646588 (lncRNA DLEU1) (**Supplementary Figure S4C**).

**Figure 5:**
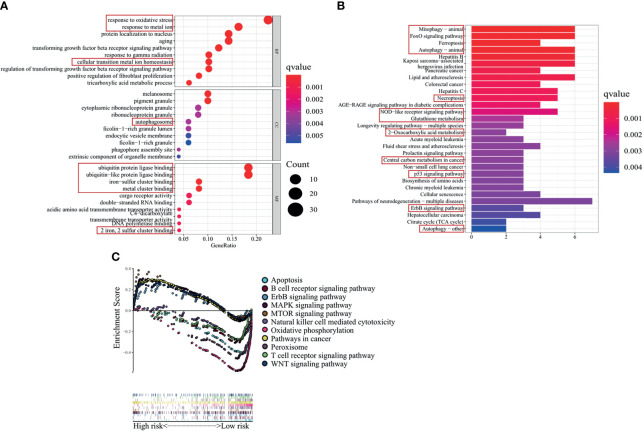
Functional and pathway enrichment analyses in The Cancer Genome Atlas—stomach adenocarcinoma (TCGA-STAD) cohort. **(A)** GO and **(B)** KEGG enrichment analyses of ferroptosis-related genes differentially expressed in the high- and low-risk groups of TCGA-STAD cohort. The main focus is on the functions and pathways related to ferroptosis and tumor progression (framed by red boxes). **(C)** GSEA based on ferroptosis-related ncRNA signature. GO, Gene Ontology; KEGG, Kyoto Encyclopedia of Genes and Genomes; GSEA, gene set enrichment analysis.

The GO enrichment analysis of ferroptosis-related hub genes was performed. The results suggested that the TCGA-STAD cohort was enriched in functions related to oxidative stress, metal iron ion, autophagy, ubiquitin, antioxidant, peroxidase, and oxidoreductase (response to oxidative stress, response to metal ion, cellular transition metal ion homeostasis, autophagosome, ubiquitin protein ligase binding, ubiquitin-like protein ligase binding, iron−sulfur cluster binding, metal cluster binding, and two-iron and two-sulfur cluster binding) (**Figure 5A**). The KEGG enrichment analysis suggested that the TCGA-STAD cohort was enriched in mitophagy—animal, forkhead box O (FoxO) signaling pathway, ferroptosis, autophagy—animal, necroptosis, NOD-like receptor signaling pathway, glutathione metabolism, 2-oxocarboxylic acid metabolism, central carbon metabolism in cancer, p53 signaling pathway, erythroblastic oncogene B (ErbB) signaling pathway, and autophagy—other (**Figure 5B**).

As shown in **Figure 5C**, GSEA between the high- and low-risk groups suggested that the high-risk group was enriched in ErbB signaling pathway, MAPK signaling pathway, mTOR signaling pathway, pathways in cancers, and WNT signaling pathway, and the low-risk group was enriched in apoptosis, B-cell receptor signaling pathway, natural killer cell-mediated cytotoxicity, oxidative phosphorylation, peroxisome, and T-cell receptor signaling pathway.

In general, ferroptosis-related hub genes closely related to the risk signature, and they had a strong protein interaction and expression correlation; they also had a strong co-expression relationship with five ncRNAs. CRYAB was a core gene, which regulated multiple ferroptosis-related hub genes, and protein interaction was found among the ferroptosis-related hub genes. CRYAB was also co-expressed with ferroptosis-related ncRNAs DLEU1 and LOC646588. The GO enrichment analyses indicated that ferroptosis-related genes differentially expressed in the high- and low-risk groups were mainly enriched in functions related to oxidative stress, iron ion, autophagy, and ubiquitination. The KEGG enrichment analyses indicated that they were mainly enriched in ferroptosis-related metabolic pathways and cancer-related pathway. GSEA was enriched in apoptosis-related pathways, tumor occurrence and development-related pathways, oxidation-related pathways, and immune-related pathways.

### Risk Signature-Based Immune Cell Infiltration, Immune-Related Pathways, TME, and Immune Checkpoint Gene Analyses

The difference in the risk score was analyzed for each cluster to combine clusters with ferroptosis-related ncRNA signature and jointly analyze immune cell infiltration and TME. As shown in **Supplementary Figure S5A**, cluster 4 had the highest risk score, and cluster 6 had the lowest risk score. Differences in risk scores were found among most clusters (*P* < 0.05). In **Supplementary Figure S5A**, combined with the heat map of the clinical feature correlation analysis, clusters 1, 2, and 6 represented the low-risk group, and clusters 3, 4, 5, and 7 represented the high-risk group. As shown in **Supplementary Figure S5B**, the ESTIMATE score correlated negatively with tumor purity. **Supplementary Figure S5C** shows that the risk score was notably higher for samples with a high ESTIMATE score than for those with a low ESTIMATE score (*P* = 0.00014). The stromal score, immune score, and ESTIMATE score of clusters 4 and 5 were higher than those of other clusters, and the stromal score, immune score, and ESTIMATE score of cluster 6 were the lowest (**Supplementary Figures S5D, F**). This result again confirmed that the ESTIMATE score of clusters 4 and 5, representing the high-risk group, was the highest, and the estimated score of clusters 4 and 6, representing the low-risk group, was the lowest.

**Figure 6:**
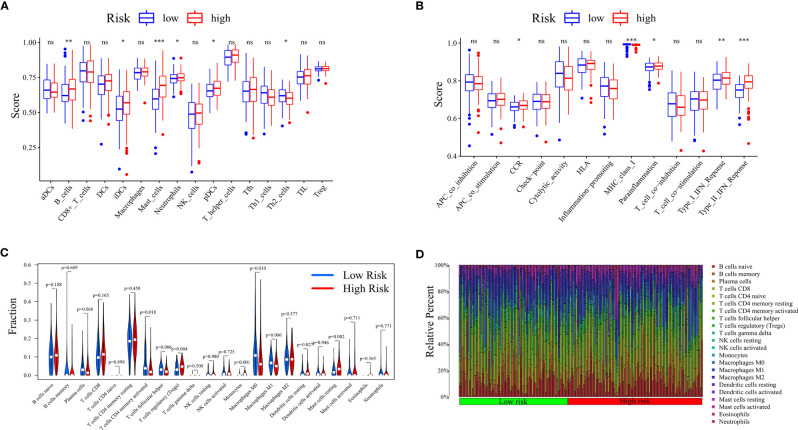
Analysis of immune cell infiltration between high- and low-risk groups. Risk differential analysis of **(A)** immune cells and **(B)** immune-related pathways based on ssGSEA. **(C)** Violin plots showing the content difference of multiple immune cells between high- and low-risk groups based on the CIBERSORT algorithm. **(D)** Histogram showing the composition of the immune cells of each sample in the high- and low-risk groups based on the CIBERSORT algorithm. ssGSEA, single-sample gene set enrichment analysis. ns, not significat; *P < 0.05; **P < 0.01; ***P < 0.001.

The score difference analyses of 16 immune cells in the high- and low-risk groups based on ssGSEA suggested that the scores of B cells (^**^ *P* < 0.01), iDCs (^*^ *P* < 0.05), mast cells (^***^ *P* < 0.001), neutrophils (^*^ *P* < 0.05), and pDCs (^*^ *P* < 0.05) were significantly higher in the high-risk group than in the low-risk group. The score of Th2 cells (^*^ *P* < 0.05) was significantly lower in the high-risk group than in the low-risk group (**Figure 6A**). The score differential analysis of 13 immune-related pathways suggested that the scores of chemokines and chemokine receptors (CCR; ^*^ *P* < 0.05), parainflammation (^*^ *P* < 0.05), type I interferon (IFN) response (^**^ *P* < 0.01), and type II IFN response (^***^ *P* < 0.001) were significantly higher in the high-risk group than in the low-risk group. The score of major histocompatibility complex (MHC) class I (^***^ *P* < 0.001) was significantly lower in the high-risk group than in the low-risk group (**Figure 6B**).

**Figure 7:**
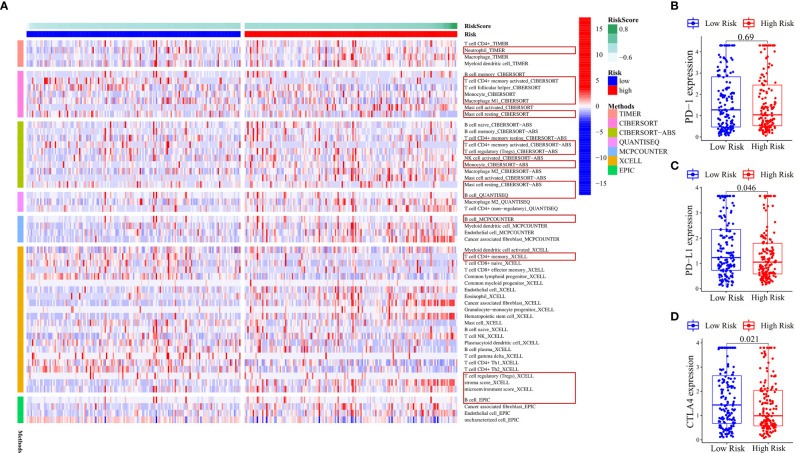
Various algorithms verified the results of immune cell infiltration and the expression of immune checkpoints in the ferroptosis-related ncRNA signature. **(A)** Heat map showing the expression difference of each immune cell based on different algorithms in the high- and low-risk groups. The red box indicates that these immune cells can verify the above-mentioned conclusions. **(B–D)** Differences in the expression of PD-1, PD-L1, and CTLA4 between high- and low-risk groups. The values in the figure are *P*-values.

As shown in **Figure 6C**, the results of immune cell infiltration obtained by the CIBERPORT algorithm showed that the contents of activated memory CD4 T cells (*P* = 0.018), T follicular helper cells (*P* = 0.006), M0 macrophages (*P* = 0.010), and M1 macrophages (*P* = 0.006) were significantly lower in the high-risk group than in the low-risk group. T-cell regulatory (Tregs) (*P* = 0.004), monocytes (*P* < 0.001), dendritic cell resting (*P* = 0.027), and Mast cell resting (*P* = 0.002) were significantly higher in the high-risk group than in the low-risk group. The immune cell histogram of the TCGA-STAD cohort could intuitively see the relative percentage of immune cells in each sample (**Figure 6D**). The number of samples with immune cell infiltration *P*-value less than 0.05 in the high-risk group was greater than that in the low-risk group. Furthermore, the Spearman correlation analysis showed that activated memory CD4 T cells (*R* = −0.22, *P* = 0.0047), T follicular helper cells (*R* = −0.3, *P* = 7.2e-05), M0 macrophages (*R* = −0.29, *P* = 0.00018), and M1 macrophages (*R* = −0.21, *P* = 0.0065) correlated negatively with the risk score, and Tregs (*R* = 0.19, *P* = 0.014), monocytes (*R* = 0.36, *P* = 1.5e-06), and Mast cell resting (*R* = 0.25, *P* = 9e-04) correlated positively with the risk score (**Supplementary Figures S6A–G**). Subsequently, the differential analysis of immune cells in clusters showed that the contents of activated memory CD4 T cells (**Supplementary Figure S6H**), T follicular helper cells (**Supplementary Figure S6I**), M0 macrophages (**Supplementary Figure S6L**), and M1 macrophages (**Supplementary Figure S6M**) of clusters 4 and 5, representing the high-risk group, were lower than those of Cluster 6, representing the low-risk group, and the contents of Tregs (**Supplementary Figure S6J**), monocytes (**Supplementary Figure S6K**), and resting mast cells (**Supplementary Figure S6N**) of clusters 4 and 5, representing the high-risk groups, were higher than those of cluster 6, representing the low-risk group. The TME, results of ssGSEA and CIBERSORT, and **Figure 7A** were combined for validation. We concluded that the contents of neutrophils, monocytes, resting mast cells, Tregs, and B cells were significantly higher in the high-risk group than in the low-risk group. Moreover, the contents of activated memory CD4 T cells and T follicular helper cells were significantly lower in the high-risk group than in the low-risk group. The stromal score and ESTIMATE score were higher in the high-risk group than in the low-risk group. The tumor purity was lower in the high-risk group.

No difference was found in the expression of immune checkpoint gene PD-1 (**Figure 7B**) between the high- and low-risk groups. The expression of PD-L1 (*P* = 0.046) (**Figure 7C**) and CTLA4 (*P* = 0.021) (**Figure 7D**) was higher in the low-risk group than in the high-risk group. According to **Supplementary Figures S1B–D**, the expression of three immune checkpoint genes in clusters 1 and 2 was higher than that in other clusters. Clusters 1, 2, and 6 were representatives of the low-risk groups, but cluster 6 was an exception. The expression of three immune checkpoint genes in cluster 6 was not high. These results indicated that the expression of the three immune checkpoint genes had little relationship with risk signature; it was only slightly higher in the low-risk group. It was more closely related to clusters. The three immune checkpoint genes were highly expressed in clusters 1 and 2. The heat map of immune-related genes downregulating the immune response from the TIP database (**Supplementary Figure S7A**) showed that NOS1, TIMD4, TGFB1, CXCL12, CCL2, NOS3, EDNRB, and VSIR were upregulated in the high-risk group and CCL28, NOS2, TNFRSF14, VEGFA, SMC3, DNMT1, EZH2, MICB, CTLA4, and IDO1 were upregulated in the low-risk group.

The analysis of tumor microenvironment, immune cell infiltration, and immune checkpoint genes revealed the mechanism of tumor immune microenvironment in gastric adenocarcinoma based on five-ncRNA risk signature and consensus clustering.

### Immunotherapy, Drug Screening, and Drug IC_50_ Prediction Based on Ferroptosis-Related ncRNA Signature to Improve the Survival of Patients With Gastric Adenocarcinoma

**Figure 8:**
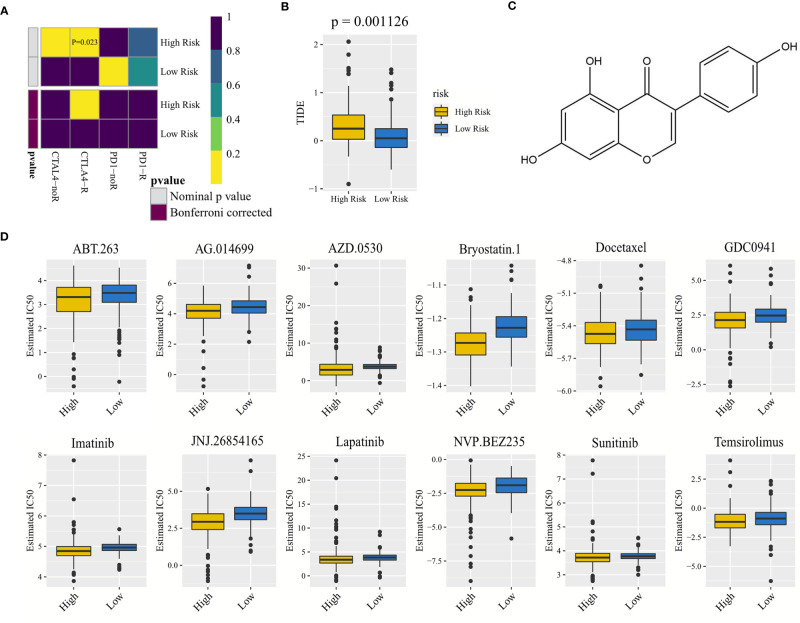
Correlated with anti-CTLA4 and anti-PD-1 immunotherapy, drug screening, and drug IC_50_ prediction. **(A)** Heat map exploring the response of anti-CTLA4 and anti-PD-1 immunotherapy for gastric adenocarcinoma in the high- and low-risk groups. CTLA4-noR, anti-CTLA4 immunotherapy did not respond; CTLA4-R, anti-CTLA4 immunotherapy responded, and so on. Including nominal *P*-value and Bonferroni corrected *P*-value. **(B)** Difference in the TIDE score between the high- and low-risk groups. **(C)** Structure of the small-molecule drug genistein. Genistein is a natural aromatic oxygenated heterocyclic compound, which can inhibit the expression of risk-model-related genes and lead to apoptosis of gastric cancer cells. **(D)** Differences in IC_50_, estimated between the high- and low-risk groups, of 12 drugs that were conducive to reducing the risk of gastric adenocarcinoma and improving survival. TIDE, tumor immune dysfunction and exclusion; IC_50_, half-maximal inhibitory concentration.

In terms of nominal *P*-value, anti-CTLA4 immunotherapy was more likely to respond in the high-risk group (*P* = 0.023), and its Bonferroni-corrected *P*-value was less than that in other cases (**Figure 8A**). The TIDE score was lower in the low-risk group than in the high-risk group (*P* = 0.001126), indicating that the response of the low-risk group to immune checkpoint inhibitors was greater compared with the high-risk group (**Figure 8B**).

A total of 134 drugs with a *P*-value <0.05 were screened using the CMAP drug screening of differentially expressed genes in the high- and low-risk groups. Three common drugs with an enrichment value less than 0 were selected to reduce patient risk, namely, genistein (*P* = 0.00793, enrichment = −0.391) (**Figure 8C**), guanabenz (*P* = 0.04047, enrichment = −0.557) (**Supplementary Figure S7B**), and betulinic acid (*P* = 0.02274, enrichment = −0.681) (**Supplementary Figure S7C**).

Ridge regression models were constructed to predict the IC_50_ of drugs, and 12 drugs with a *P*-value less than 0.05 were found. Their IC_50_ values in the low-risk group were higher than those in the high-risk group (**Figure 8D**), indicating that their antitumor efficacy was better in the high-risk group than in the low-risk group, and the antitumor efficacy of docetaxel was the best among the 12 drugs due to its lowest IC_50_ value.

## Discussion

Since few ferroptosis-related ncRNAs were shared by the TCGA-STAD and GSE84426 cohorts, we screened five optimal ferroptosis-related ncRNAs with the prognostic value in gastric adenocarcinoma: lncRNA TMEM105 (ENSG00000185332), lncRNA PVT1 (ENSG00000249859), ncRNA LOC646588 (ENSG00000223561), ncRNA FLJ22447 (ENSG00000232774), and lncRNA DLEU1 (ENSG00000176124). Extensive studies have demonstrated that lncRNA PVT1 was a carcinogenic RNA (14). Li et al. concluded that the expression of lncRNA PVT1 highly correlated with the poor prognoses of patients with gastric adenocarcinoma (15). Lu et al. concluded that PVT1 regulated ferroptosis mediated by miR-214 (16). LncRNA DLEU1 was also considered a potential carcinogenic RNA in gastric adenocarcinoma. Song et al. confirmed that lncRNA DLEU1 was abnormally elevated in gastric cancer (17). LncRNA DLEU1 predicted a poor prognosis of gastric cancer and promoted cell proliferation through the apparent inhibition of KLF2 (18). The present study also suggested that the expression of PVT1 and DLEU1 in tumor tissues was notably higher than that in normal tissues (18–21). A positive regulatory relationship existed between lncRNA DLEU1 and lncRNA PVT1, and the effects of lncRNA DLEU1 and lncRNA PVT1 on gastric adenocarcinoma were similar, both of which were highly expressed in tumor tissues. However, the hazard ratios of lncRNA PVT1 and lncRNA DLEU1 were less than 1, indicating that they were protective factors for gastric adenocarcinoma. The expression of lncRNA PVT1 was concentrated in the low-risk group and cluster 1, representing the low-risk group, and that of lncRNA DLEU1 was concentrated in the low-risk group and clusters 1, 2, and 6, representing the low-risk group. This was because lncRNA DLEU1 and lncRNA PVT1 were indeed carcinogenic RNA, but their expression was inhibited in the high-risk group. As the condition of patients in the high-risk group deteriorated to a certain extent, the expression of lncRNA DLEU1 and lncRNA PVT1 was inhibited. However, even if their expression decreased, the prognosis still could not be improved. They appeared to be protective factors for gastric adenocarcinoma, but they were still hazard factors. In addition, a great difference was found in the expression of lncRNA DLEU1 and lncRNA PVT1 between gastric adenocarcinoma tumor tissues and normal tissues, but little difference was found in the expression level in tumor tissues, resulting in the illusion that a high expression of lncRNA DLEU1 and lncRNA PVT1 could reduce the risk of patients in tumor tissues.

Differently from lncRNA DLEU1 and lncRNA PVT1, the expression of ncRNA LOC646588 was downregulated in tumor tissues *versus* normal tissues, but the hazard ratio of ncRNA LOC646588 was greater than 1, and the *P*-value was less than 0.01. ncRNA LOC646588 was mainly highly expressed in the high-risk group and clusters 4 and 5, representing the high-risk group, which was negatively regulated by lncRNA DLEU1. In this study, we still inferred that ncRNA LOC646588 could predict the poor prognosis of gastric adenocarcinoma. It is an RNA that plays roles in various cases. Without gastric adenocarcinoma, even if ncRNA LOC646588 was highly expressed, it did not play its role. Once patients suffer from gastric adenocarcinoma, the high expression of ncRNA LOC646588 worsens the prognoses of patients. We believed that attention should be paid to the results of the synthesis of multiple ncRNAs and not just a single ncRNA. In addition, ncRNA FLJ22447 and lncRNA TMEM105 were upregulated in tumor tissues *versus* normal tissues, and their hazard ratio was greater than 1, which was mainly highly expressed in the high-risk group and clusters representing the high-risk group. We have reasons to infer that ncRNA FLJ22447 and lncRNA TMEM105 were carcinogenic ncRNA in gastric adenocarcinoma. The expression of PD-1 (gene of PD-1) correlated negatively with the expression of lncRNA TMEM105. ncRNA may simultaneously contain a tumor-suppressive effect and carcinogenic effect, depending on which effect is dominant.

Promoting intensive ferroptosis of gastric adenocarcinoma cells is conducive to delaying tumor development. When the gastric gland tissue changes from normal to tumor, the gene promoting cell ferroptosis is highly expressed, resulting in a potentially high ferroptosis level as a whole so as to inhibit the proliferation of tumor cells. The correlation analysis between risk and clinical characteristics confirmed that the FPI of the high-risk group was lower than that of the low-risk group, indicating that the high-risk group may have a lower level of ferroptosis (6), resulting in poor prognoses of patients in the high-risk group. From normal tissue to tumor tissue, the potential level of ferroptosis was greatly improved. However, in the high-risk group, the expression of ferroptosis-related genes that promoted the increase in the ferroptosis level was insufficient, resulting in the reduction of potential ferroptosis level and poor prognosis in the high-risk group.

The results of this study showed that the TMB was generally higher in the low-risk group. The greater the TMB, the more the DNA mutations and the more the production of candidate peptides, resulting in a higher possibility that the new antigen would be successfully recognized by the immune system and the prognoses of patients with gastric adenocarcinoma would be better (22). With these characteristics, the TMB has become a potential biomarker for immunotherapy of gastric adenocarcinoma. However, some studies pointed out that TP53 mutation played a negative role in the antitumor immunity of gastric adenocarcinoma (23). We prefer the first to explain the results of this study, that is, the higher the TMB, the better the prognosis of gastric adenocarcinoma.

We developed a nomogram containing excellent independent prognostic factors, which could accurately predict the prognoses of patients with gastric adenocarcinoma. The DCA showed that, in most cases, risk signature had the highest accuracy in predicting the survival of patients with gastric adenocarcinoma.

This study suggested that CRYAB, GABARAPL1, TGFBR1, and CAV1 were the ferroptosis-related hub genes highly related to the ferroptosis-related ncRNA signature. The high expression of CRYAB may lead to poor prognoses in patients with gastric cancer (24). GABARAPL1 was closely related to autophagy and promoted the growth of a variety of cancers (25). TGFBR1 was associated with poor survival in patients with gastric cancer (26). The expression of CAV1 in gastric cancer–associated fibroblasts and their corresponding inflammation-associated fibroblasts was used as a potential biomarker of gastric cancer progression (27). With CRYAB as the center, GABARAPL1, TGFBR1, and CAV1 all had a positive regulatory relationship with CRYAB, indicating that these hub genes were the whole of ferroptosis-related oncogenes. This whole was linked with ferroptosis-related oncogenic ncRNAs through the co-expression of GABARAPL1, TGFBR1, and CAV1, forming a complex carcinogenic network of ferroptosis-related hub genes and ferroptosis-related ncRNAs. It showed that the co-expression network based on ferroptosis-related ncRNA signature was closely related to the occurrence and development of gastric adenocarcinoma, which led to the poor prognoses of patients. This result demonstrated that ncRNA-regulated ferroptosis-related genes were also a potential biomarker of gastric adenocarcinoma.

The GO enrichment analyses of ferroptosis-related genes differentially expressed in high- and low-risk groups mainly enriched the functions related to oxidative stress, iron ion, and autophagy, which were highly correlated with ferroptosis. First, ferroptosis is characterized by the accumulation of lipid peroxidation products and lethal reactive oxygen species from iron metabolism (28). The demand for iron gradually increases with the cancerization of cells. This iron dependence makes cancer cells more vulnerable to iron-catalyzed necrosis. In addition, oxygen species in ferroptosis may trigger autophagy, which may enhance cellular ferroptosis (29). Finally, ubiquitination is closely related to the pathogenesis of tumor. Ubiquitin protein ligase catalyzes the posttranslational modification of abnormal proteins, which may be a new treatment (30). The risk differential genes were found to have a variety of functions to induce ferroptosis and cancer.

The KEGG-enriched pathways closely related to ferroptosis and gastric adenocarcinoma. First, glutathione exists widely and has an important antioxidant effect, which inhibits ferroptosis; however, glutathione also has an antitumor effect. The loss of glutathione peroxidase 4 activity has long been a major feature of ferroptosis (31). Second, the FoxO signaling pathway is involved in many physiological events of cells, such as apoptosis, cell cycle control, and antioxidant stress (32). Third, the NOD-like receptor signaling pathway may promote tumor angiogenesis and increase cancer risk. The low expression of ANGPT2 also affects the level of related proteins in this pathway, thus inhibiting the metastasis of gastric cancer cells (33). In addition, the mTOR signaling pathway exists widely and controls the proliferation and metabolism of cancer cells (34). The autophagy-related pathway in gastric cancer is related to not only the P53 pathway but also the mTOR pathway. It is also possible to promote autophagy-dependent ferroptosis by activating the AMPK–mTOR pathway (35). Finally, the ErbB signaling pathway is related to the angiogenesis of tumor cells (35), indicating that it may be a therapeutic target for a variety of cancers. Various signal pathways are more or less connected, and they are inseparable from tumor and ferroptosis.

GSEA demonstrated that the signal pathways leading to cancer were enriched in the high-risk group, and the signal pathways related to ferroptosis and immunity were enriched in the low-risk group. The activation of enriched signal pathways in the high-risk group indicated that the prognoses of patients with gastric adenocarcinoma were poor, while the enriched pathways in the low-risk group were conducive to promoting ferroptosis and tumor immunity and reducing the risk of patients with gastric adenocarcinoma.

Gastric adenocarcinoma tissue contains not only gastric adenocarcinoma cells but also nontumor cells related to gastric adenocarcinoma, such as stromal cells and immune cells, which reduce the purity of the tumor. This study showed that the lower the purity of the tumor, the higher the risk score and the worse the prognoses of patients, indicating that stromal cells still played a role in nontumor cells, and the role of immune cells was not enough. The content of immune cells in the high-risk group was far from enough to improve the prognoses of patients with gastric adenocarcinoma. As part of the tumor immune microenvironment, neutrophil infiltration increased significantly in gastric cancer, and this study showed that the neutrophil content increased in the high-risk group. Neutrophils in the high-risk group might promote the metastasis of gastric cancer (36). The most prominent feature of monocytes in gastric adenocarcinoma was that they could recognize and kill tumors (37); therefore, they were more likely to participate in effective immune response in the high-risk group to try their best to kill cancer cells. Mast cells in the high-risk group were also extensive. They promoted the formation of blood vessels and lymphatic vessels of gastric cancer and played a role in promoting cancer (38). The high content of Tregs in the high-risk group was explained by the fact that Tregs reduced the immunity of the body to gastric adenocarcinoma (39), resulting in an increase in the mortality of patients. The B cells were also concentrated in the high-risk group, which were associated with better prognoses in patients with gastric adenocarcinoma (40). In contrast, activated memory CD4 T cells and T follicular helper cells were more abundant in the low-risk group. CD4 + T cells and T follicular helper cells were likely to promote the maintenance of a protective immune response (41, 42), which was related to the good prognoses of patients with gastric adenocarcinoma in the low-risk group.

On the one hand, CCRs were related to the proliferation and migration of cancer cells for the immune-related pathways and were potential targets of immunotherapy in the future (43). Parainflammation is a state between normal homeostasis and inflammatory response. It exists widely in cancer and is related to poor prognosis (44). Studies have shown that the high concentrations of type 1 IFN could weaken the immune response (45), and type 2 IFN response might contribute to the development of gastric diseases (46). The aforementioned immune-related pathways were concentrated in the high-risk group, which was related to the poor prognosis of gastric adenocarcinoma. On the other hand, MHC class 1 was enriched in the low-risk group, indicating a good prognosis. MHC class 1 had the highest score in all immune-related pathways. The downregulation of MHC was conducive to the resistance of the tumor to immune response (47), which was the case in the high-risk group.

To obtain the detailed tumor classification of gastric adenocarcinoma and establish a new grouping system, we conducted unsupervised class discovery, and clusters and risk groups were connected as follows: clusters 1, 2, and 6 represented the low-risk group, and the other clusters represented the high-risk group. However, PD-1, PD-L1, and CTLA4 were highly expressed in clusters 1 and 2 and less expressed in cluster 6, but clusters 1, 2, and 6 all represented the low-risk group. This result showed that the expression of these three immune checkpoints might have been separated from the high- and low-risk groups and have a greater relationship with clusters. They were mainly highly expressed in clusters 1 and 2. The findings of the present study showed that lncRNA TMEM105 was highly expressed in cluster 3, and our correlation analysis showed that the expression of lncRNA TMEM105 inhibited the expression of PD-1. We were surprised to find that the expression of PD-1 in cluster 3 was really inhibited, indicating that the high expression of lncRNA TMEM105 was likely to inhibit the expression of PD-1.

For high-risk patients, the effect of anti-CTLA4 immunotherapy was better; for low-risk patients, the effect of anti-PD-1 immunotherapy was relatively good. The TIDE score was lower in the low-risk group, indicating that the immune checkpoint inhibitor immunotherapy tended to respond in the low-risk group (9, 10). The reason may be that gastric adenocarcinoma in the high-risk group was very serious or the high expression of ncRNAs in the high-risk group weakened the effect of immunotherapy. For the immune-related genes with downregulated immune function obtained in the TIP database, the high expression of NOS1, TIMD4, TGFB1, CXCL12, CCL2, NOS3, EDNRB, and VSIR in the high-risk group weakened the antitumor immunity and worsened the prognosis. The low expression of CCL28, NOS2, TNFRSF14, VEGFA, SMC3, DNMT1, EZH2, MICB, CTLA4, and IDO1 in the high-risk group improved the antitumor immune ability as much as possible. CTLA4 belonged to the latter. These immune-related genes might be the objects of immune checkpoint inhibitor immunotherapy based on the ferroptosis-related ncRNA signature.

The drugs genistein, guanabenz, and betulinic acid, screened by ferroptosis-related ncRNA signature, could reduce the risk and improve the survival of patients with gastric adenocarcinoma because they can inhibit the expression of risk-model-related genes in the high-risk groups. Genistein, a natural aromatic oxygenated heterocyclic compound, could inhibit the proliferation of a variety of cancer cells and lead to apoptosis of gastric cancer cells (48). Guanabenz, a drug for treating hypertension, could prevent tumor cell proliferation and reduce tumor invasiveness (49). Betulinic acid is a complex pentacyclic triester with a strong antitumor activity, which could inhibit the proliferation and migration of gastric cancer cells (50). The 12 anti-gastric-adenocarcinoma drugs [ABT.263 (navitoclax), AG.014699 (rucaparib), AZD.0530 (saracatinib), bryostatin.1, docetaxel, GDC0941 (pictilisib), imatinib, JNJ.26854165 (serdemetan), lapatinib, NVP.BEZ235 (dactolisib), sunitinib, and temsirolimus] screened by the GDSC database performed better in the high-risk group than in the low-risk group, which provided a second choice to reduce the risk of patients with gastric adenocarcinoma. Docetaxel had an excellent anticancer effect without considering other factors.

Briefly, we constructed a ferroptosis-related oncogenic ncRNA signature. No clinical characteristic was superior to it in predicting the prognoses of patients with gastric adenocarcinoma. The high-risk group had low tumor purity, low FPI, few mutations, more enrichment of carcinogenic pathways, more basic immune cells, low expression of immune checkpoints, and a difficult response to immune checkpoint inhibitor immunotherapy. Moreover, the low-risk group had high tumor purity, high FPI, more mutations, more enrichment of immune-related pathways, more CD4+ T-related cells, higher expression of immune checkpoints, and easy response to immune checkpoint inhibitor immunotherapy. The nomogram could independently predict the survival of patients with gastric adenocarcinoma, and the selected anticancer drugs could prospectively reduce the risk of patients with gastric adenocarcinoma. In addition, the ferroptosis-related mRNA–ncRNA co-expression network may be a biomarker to predict the poor prognoses of patients with gastric adenocarcinoma, and the selected immune-related genes were potential immune checkpoints in immune checkpoint inhibitor immunotherapy, and lncRNA TMEM105 was also a potential PD-1 inhibitor.

Many studies have also reported that ferroptosis-related noncoding RNA signature is a prognostic biomarker (51–53). The advantage of our research is that we use the data of the TCGA database to build the signature and also use the data of the GEO database for external verification. In addition, our study introduced clinical characteristics, such as FPI, TMB, and TIDE, and analyzed immunotherapy and drug prediction. Immunotherapy and the screened drugs are conducive to improve the survival of patients with gastric adenocarcinoma.

This study had some limitations. First, this study was limited to mining and analyzing public databases. Second, the detailed mechanism of the five ferroptosis-related ncRNAs in the process of antitumor immunity was not clear.

## Data Availability Statement

The original contributions presented in the study are included in the article/**Supplementary Material**. Further inquiries can be directed to the corresponding author.

## Author Contributions

LL conceived and designed the study, reviewed the paper, and provided comments. XC, ZZ, and XY performed data mining and analysis. XC, ZZ, LL, and XL wrote the manuscript. All authors contributed to the article and approved the submitted version.

## Funding

This project was supported by the Administration of Traditional Chinese Medicine of Guangdong Province (20201180), the Administration of Traditional Chinese Medicine of Guangdong Province (20211223), the Science and Technology Special Project of Zhanjiang (2019A01009), the Basic and Applied Basic Research Program of Guangdong Province (2019A1515110201), the Program of Department of Natural Resources of Guangdong Province [nos. GDNRC (2020)038 and (2021)53], and the Discipline Construction Project of Guangdong Medical University (4SG21004G).

## Conflict of Interest

The authors declare that the research was conducted in the absence of any commercial or financial relationships that could be construed as a potential conflict of interest.

## Publisher’s Note

All claims expressed in this article are solely those of the authors and do not necessarily represent those of their affiliated organizations, or those of the publisher, the editors and the reviewers. Any product that may be evaluated in this article, or claim that may be made by its manufacturer, is not guaranteed or endorsed by the publisher.

## Supplementary Material

Supplementary Figure 1 Kaplan–Maier survival curve analysis of **(A)** ncRNA FLJ22447, **(B)** ncRNA LOC646588, **(C)** lncRNA TMEM105, **(D)** lncRNA PVT1, and **(E)** lncRNA DLEU1.

Supplementary Figure 2 Consensus clustering and independent prognostic factor analyses. **(A)** Consensus clustering plots further verify that the optimal number of clusters is seven, not others, indicating that it is reasonable to divide into seven clusters. **(B–D)** Expression of PD-1, PD-L1, and CTLA4 in different clusters. **(E)** Univariate Cox analysis based on clinical characteristics in The Cancer Genome Atlas—stomach adenocarcinoma cohort. **(F)** Univariate Cox analysis based on clinical characteristics in the GSE84426 cohort. **(G)** Multivariate Cox analysis based on clinical characteristics in the GSE84426 cohort. LASSO, least absolute shrinkage and selection operator; PCA, principal component analysis.

Supplementary Figure 3 DCA, correlation between risk and FPI, correlation between risk, and TMB. **(A)** Net reduction DCA of The Cancer Genome Atlas—stomach adenocarcinoma cohort. “Combined” represents the model jointly constructed by all clinical characteristics in the figure. **(B)** Net benefit DCA of the GSE84426 cohort. **(C)** Net reduction DCA of the GSE84426 cohort. **(D)** FPI difference between normal and tumor tissues in stomach adenocarcinoma. **(E)** Analysis of FPI difference between high- and low-risk groups. **(F)** Analysis of TMB difference between high- and low-risk groups. DCA, decision curve analysis; FPI, ferroptosis potential index; TMB, tumor mutation burden.

Supplementary Figure 4 Construction of hub gene oncogenic interaction network. **(A)** Protein–protein interaction network of hub genes. **(B)** Expression correlation network of hub genes. **(C)** Co-expression network of hub genes and five ncRNAs.

Supplementary Figure 5 Tumor microenvironment analyses. **(A)** Analysis of risk score difference of clusters. **(B)** Correlation analysis between ESTIMATE score and tumor purity, for example, sample TCGA-3M-AB47. **(C)** Analysis of risk score difference of high- and low-ESTIMATE-score groups. **(D–F)** Correlation analysis of stromal score, immune score, and ESTIMATE score of clusters. ESTIMATE, an algorithm for reflecting tumor purity.

Supplementary Figure 6 Analyses of immune cell infiltration based on risk score and clusters. **(A–G)** Correlation analyses between risk score and the content of seven immune cells with significant results. **(H–N)** Analyses on the content difference of seven immune cells based on clusters.

Supplementary Figure 7 Differential expression analysis of immune genes based on high- and low-risk groups and significant drugs from CMAP analysis. **(A)** Heat map for immune checkpoint genes generated by comparison of the high-risk group *versus* the low-risk group. **(B)** Guanabenz. **(C)** Betulinic acid. CMAP, connectivity map. **P* < 0.05; ***P* < 0.01; ****P* < 0.001.
