# Supplementary figures and images for: The Ferroptosis-Related Noncoding RNA Signature as a Novel Prognostic Biomarker in the Tumor Microenvironment, Immunotherapy, and Drug Screening of Gastric Adenocarcinoma
Source: Front Oncol. 2021 Nov 1;11:778557. doi: 10.3389/fonc.2021.778557 (PMC8591298; doi:10.3389/fonc.2021.778557)

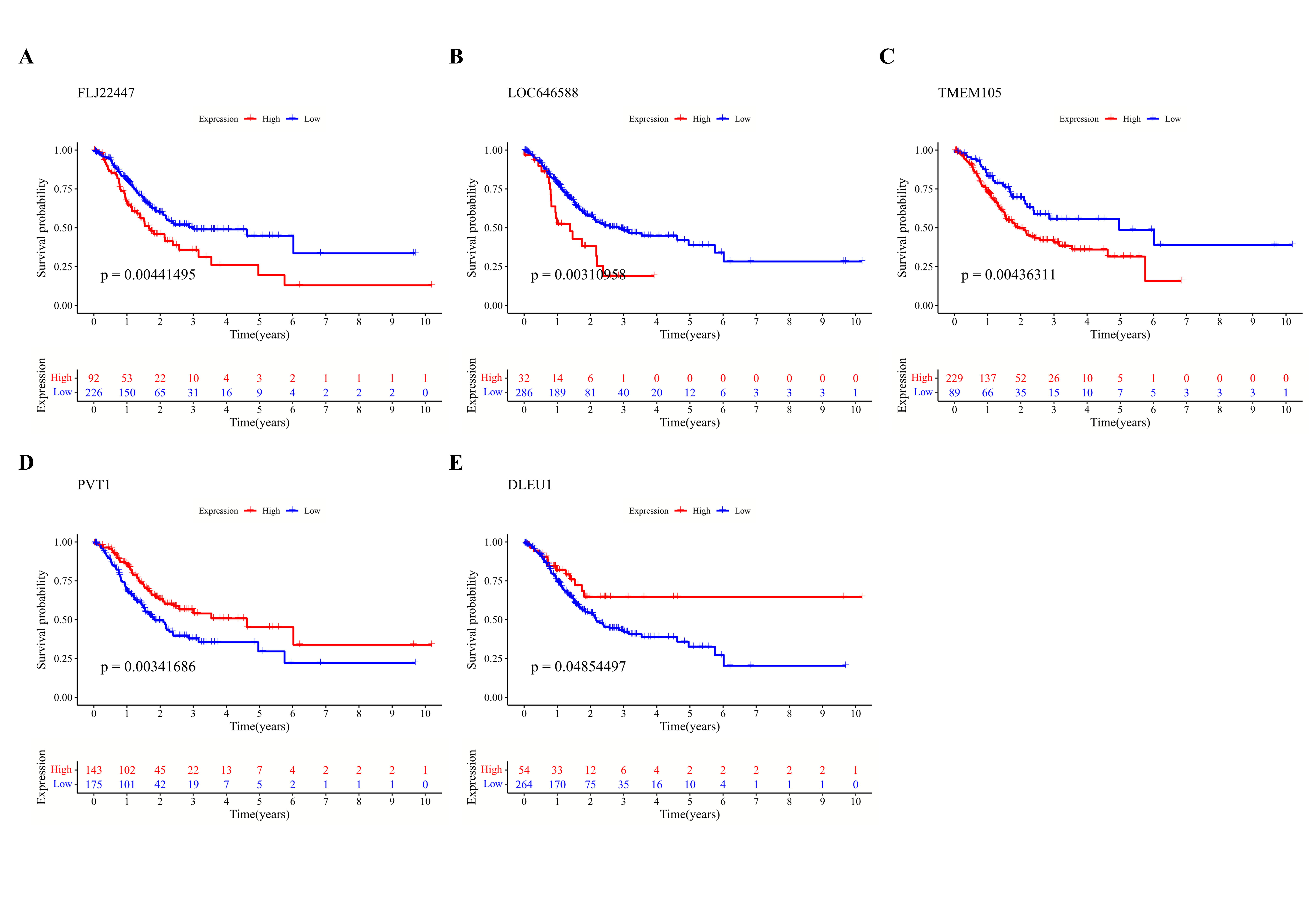

Supplement: Supplementary Figure 1 — Kaplan–Maier survival curve analysis of (A) ncRNA FLJ22447, (B) ncRNA LOC646588, (C) lncRNA TMEM105, (D) lncRNA PVT1, and (E) lncRNA DLEU1. [file Image_1.tiff]

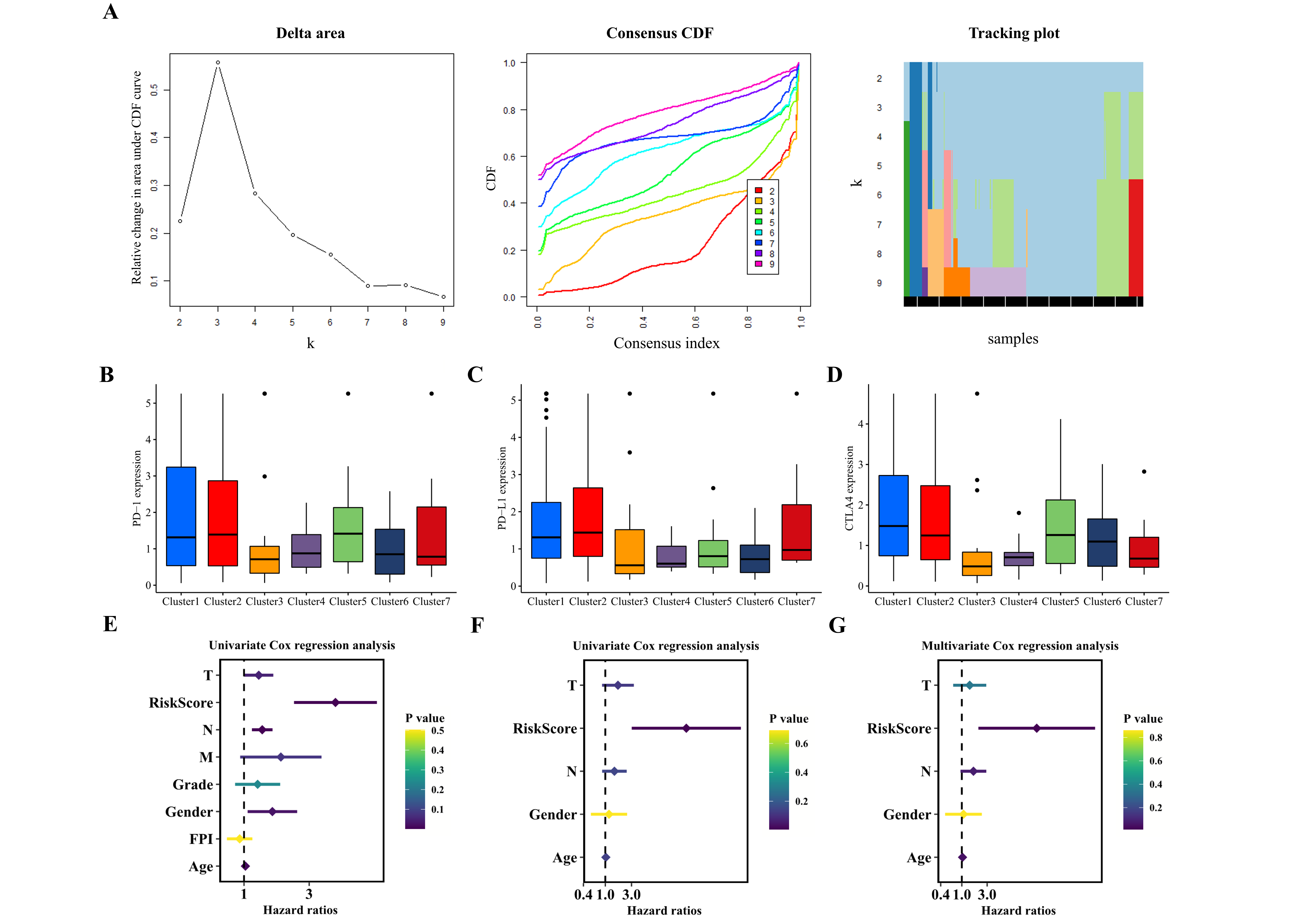

Supplement: Supplementary Figure 2 — Consensus clustering and independent prognostic factor analyses. (A) Consensus clustering plots further verify that the optimal number of clusters is seven, not others, indicating that it is reasonable to divide into seven clusters. (B–D) Expression of PD-1, PD-L1, and CTLA4 in different clusters. (E) Univariate Cox analysis based on clinical characteristics in The Cancer Genome Atlas—stomach adenocarcinoma cohort. (F) Univariate Cox analysis based on clinical characteristics in the GSE84426 cohort. (G) Multivariate Cox analysis based on clinical characteristics in the GSE84426 cohort. LASSO, least absolute shrinkage and selection operator; PCA, principal component analysis. [file Image_2.tiff]

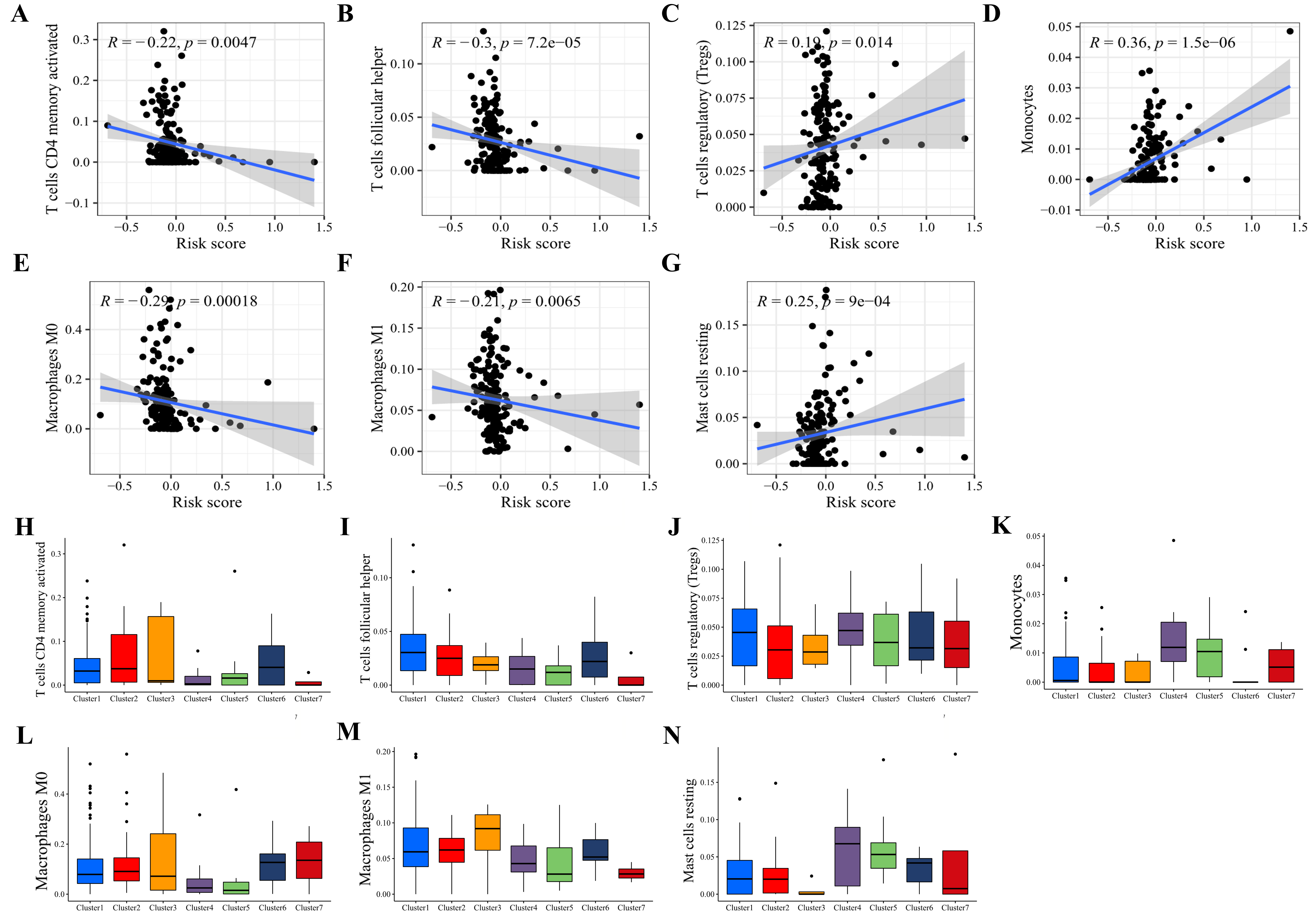

Supplement: Supplementary Figure 6 — Analyses of immune cell infiltration based on risk score and clusters. (A–G) Correlation analyses between risk score and the content of seven immune cells with significant results. (H–N) Analyses on the content difference of seven immune cells based on clusters. [file Image_6.tiff]

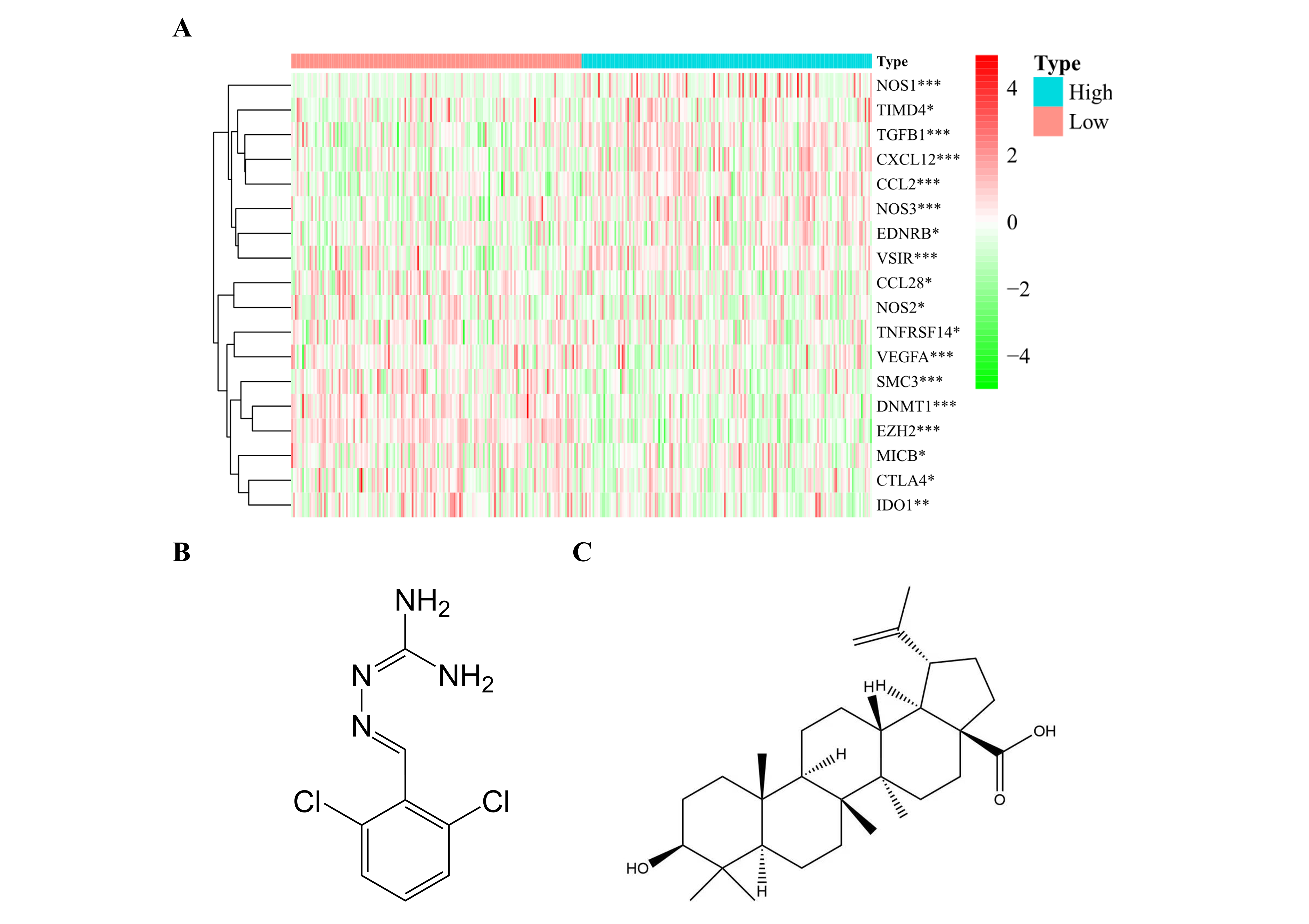

Supplement: Supplementary Figure 7 — Differential expression analysis of immune genes based on high- and low-risk groups and significant drugs from CMAP analysis. (A) Heat map for immune checkpoint genes generated by comparison of the high-risk group versus the low-risk group. (B) Guanabenz. (C) Betulinic acid. CMAP, connectivity map. *P < 0.05; **P < 0.01; ***P < 0.001. [file Image_7.tiff]
